# Nutraceutical and Antitumoral Potential of *Scenedesmus* sp. in In Vitro and In Vivo Models

**DOI:** 10.3390/foods15020186

**Published:** 2026-01-06

**Authors:** Diego Fonseca-Rivera, Diana Elia Caballero-Hernández, Patricia Tamez-Guerra, Ricardo Romero-Arguelles, Joel Horacio Elizondo-Luevano, Diana Laura Clark-Perez, Celia Maria Quiñones-Flores, Alva Rocio Castillo-Gonzalez, Ricardo Gomez-Flores, César Iván Romo-Sáenz

**Affiliations:** 1 Department of Microbiology and Immunology, School of Biological Sciences, Autonomous University of Nuevo Leon, San Nicolas de los Garza 66455, Nuevo Leon, Mexico; diego.fonsecarvr@uanl.edu.mx (D.F.-R.); diana.caballerohr@uanl.edu.mx (D.E.C.-H.); patricia.tamezgr@uanl.edu.mx (P.T.-G.); ricardoromeroarguelles@gmail.com (R.R.-A.); joel.elizondolv@uanl.edu.mx (J.H.E.-L.); diana.clarkp@uanl.edu.mx (D.L.C.-P.); 2 School of Medicine and Biomedical Sciences, Autonomous University of Chihuahua, Chihuahua 31109, Chihuahua, Mexico; cquinonezf@uach.mx (C.M.Q.-F.); acastillo@uach.mx (A.R.C.-G.)

**Keywords:** antioxidant, *Scenedesmus* sp., tumoral, nutraceutical potential, microalgae, antitumor activity, bioactive metabolites

## Abstract

Microalgae are increasingly recognized as valuable dietary supplements due to their rich nutritional composition and the presence of bioactive metabolites with antioxidant, antitumor, and metabolism-modulating activities. This study evaluated the nutraceutical potential of a methanolic extract of *Scenedesmus* sp. using an integrated in vitro, in vivo, and metabolomic approach. The extract exhibited selective cytotoxicity against L5178Y-R murine lymphoma cells while showing low toxicity toward human peripheral blood mononuclear cells, indicating a favorable safety and selectivity profile. Additionally, it demonstrated moderate antioxidant activity, a significant antihemolytic effect, and no hemolytic activity even at high concentrations, supporting its hematological safety. In vivo assays showed that oral administration of doses up to 1000 mg/kg was well tolerated, with no adverse effects on body weight or hepatic biochemical markers. Treated animals displayed improved systemic antioxidant responses and enhanced glucose tolerance. Metabolomic analysis revealed a profile enriched in essential amino acids, osmolytes, organic acids, and bioactive metabolites such as β-hydroxybutyrate and betaine, compounds associated with metabolic regulation, redox balance, and epigenetic modulation. Overall, these findings highlight *Scenedesmus* sp. as a promising nutraceutical source with potential application as a complementary strategy for cancer prevention or treatment.

## 1. Introduction

Cancer continues to be one of the leading causes of death worldwide. This disease is characterized by the appearance of cells with molecular alterations that give them the ability to self-renew, differentiate, and have high tumorigenic potential [1,2]. These characteristics lead to unregulated cell proliferation, promoting the formation of neoplasms and tumor progression in various tissues [3]. One of the current challenges in cancer treatment is the pharmacological approach due to complications such as resistance, toxicity, and relapse caused by alterations in tumor cell survival pathways [4]. Despite advances in targeted therapies, such as chemotherapy and radiotherapy, these limitations continue to affect the therapeutic efficacy and clinical prognosis of cancer patients [5], constituting one of the main barriers to successful treatment. Another problem associated with the use of chemotherapeutic agents is the occurrence of adverse effects, particularly the deterioration of immune system function, which increases health risks for patients [6]. This creates a need to develop more effective therapeutic strategies and discover new agents capable of preventing disease initiation, progression, and mortality [7]. Approximately 60% of the drugs used in hematology and oncology are derived from natural sources, highlighting the need to continue evaluating the antitumor efficacy of various bioactive compounds [8,9].

In recent years, microalgae have attracted growing interest in biomedical research, particularly due to their ability to biosynthesize high-value bioactive compounds such as carotenoids, polysaccharides, vitamins, sterols, and cyclic metabolites. Several studies have shown that these compounds possess nutraceutical properties with potential antitumor effects, mainly through the induction of apoptosis in cancer cells [10]. Among edible microalgae, *Scenedesmus* sp. has been one of the most widely studied species due to its nutritional content and bioactive compounds. One of the most notable effects is its antimicrobial activity, which is related to its high content of long-chain free fatty acids, which have been shown to induce bacterial lysis, especially eicosapentaenoic acid, which is abundant in *Scenedesmus* [11]. It has also been observed that *Scenedesmus* sp. produces monosaccharides such as glucosamine, ribose, mannose, and rhamnose, which absorb heavy metals from the environment and act as antioxidants for the elimination of free radicals, with antitumor properties by reducing the viability of various cancer cell lines [12].

On the other hand, methanolic extracts obtained from this microalga have been evaluated in vitro against tumor cell lines, showing concentration-dependent cytotoxicity, with inhibitions ranging from 15% to 75%. Furthermore, at a concentration of 500 µg/mL, these extracts did not produce significant cytotoxic effects on murine lymphocytes, presenting a cytotoxicity range of only 19–26%, in contrast to tumor cells, where cytotoxicity reached values of 62–75% [13]. In vivo studies have shown that the consumption of microalgae such as *Scenedesmus* sp. has a hypoglycemic and hypolipidemic effect, which is associated with bioactive compounds such as carotenoids, phenolic compounds, polysaccharides, and unsaturated fatty acids present in this organism [14]. In this study, the main objective was to evaluate the antitumor potential of the methanolic extract of *Scenedesmus* sp. in a murine lymphoma cell line L5178Y-R, as well as to verify in vivo its antioxidant and hypoglycemic effects in a murine model with the Balb/c strain, by consuming 1000, 500, and 250 mg/kg of the extract, respectively, to determine the nutraceutical effect of the methanol extract of *Scenedemus* sp.

## 2. Materials and Methods

### 2.1. Ethical Statement

For the use of animals, the study was submitted for revision and approval by the Research Ethics and Animal Welfare Committee of the School of Biological Sciences at UANL (protocol code CEIBA-2022-009).

Written informed consent was obtained from the healthy subject who donated the blood sample for the collection of PBMC cells. The sample was collected in the Virology and Immunology Laboratory of the School of Biological Sciences at UANL.

### 2.2. Chemicals and Reagents

All chemicals and solvents used in this study were of analytical grade. The following reagents were obtained from Sigma-Aldrich^®^ (St. Louis, MO, USA): 2,2′-azino-bis(3-ethylbenzothiazoline-6-sulfonic acid) (ABTS); 2,2′-azobis(2-methylpropionamidine) dihydrochloride (AAPH); 2,2-diphenyl-1-picrylhydrazyl (DPPH); 3-(4,5-dimethylthiazol-2-yl)-2,5-diphenyltetrazolium bromide (MTT); ascorbic acid (vitamin C); chloroform (CHCl_3_); deuterochloroform (CDCl_3_); dimethyl sulfoxide (DMSO); ethyl acetate (EtOAc); potassium persulfate (K_2_S_2_O_8_); ferric chloride; Griess reagent; lipopolysaccharide (LPS) from Escherichia coli O26:B6 (smooth strain); n-hexane; methanol (MeOH); RPMI-1640 culture medium; Sephadex^® ^(Grand Island, NY, USA); LH-20; sodium bicarbonate (NaHCO_3_); sodium chloride (NaCl); sodium hydroxide (NaOH); sodium phosphate dibasic (Na_2_HPO_4_); sodium phosphate monobasic (NaH_2_PO_4_); and sulfuric acid. 4-(2-Hydroxyethyl)-1-piperazineethanesulfonic acid (HEPES) was purchased from Invitrogen™ (Waltham, MA, USA). Dulbecco’s Modified Eagle Medium (DMEM), antibiotic–antimycotic solution (1%), fetal bovine serum (FBS), and sodium bicarbonate were obtained from Gibco™ (Grand Island, NY, USA). Vincristine sulfate (VS) was acquired from Hospira Inc. (Lake Forest, IL, USA).

### 2.3. Microalgae Strain

We used a commercial strain of *Scenedesmus* sp. with registration number BS1135 (FitoMix; Cuernavaca, Morelos, Mexico). Production was performed in a 20-L bioreactor with Bristol culture medium (FitoMix) at an initial concentration of 2 × 10^6^ cells/mL [15].

### 2.4. Extract Preparation

After 20 d of stimulation under ambient conditions, the medium with the microalgae was centrifuged at 12,000 rpm for 5 min at 4 °C to obtain three grams of biomass per bioreactor, which was lyophilized and macerated for 48 h in an amber glass flask containing 250 mL of methanol, under constant stirring. The extract was filtered and concentrated in a rotary evaporator [16].

### 2.5. Phytochemical Profile

#### 2.5.1. Alkaloids

Approximately 1 mg of each extract was dissolved in 2 mL of methanol, followed by the addition of four drops of Dragendorff reagent. The reaction was considered positive when a persistent red–orange coloration was observed. The Dragendorff reagent was freshly prepared using two solutions: Solution A, consisting of 0.85 g of Bi(NO_3_)_3_ dissolved in 10 mL of acetic acid (CH_3_COOH) and 40 mL of distilled water, and Solution B, consisting of 8 g of KI dissolved in 20 mL of distilled water. The final reagent was obtained by mixing 5 mL of Solution A, 4 mL of Solution B, and 100 mL of distilled water [17].

#### 2.5.2. Carbohydrates

The presence of carbohydrates was assessed using the Molisch test. Briefly, 1 mg of each extract was treated with Molisch reagent (1% α-naphthol in ethanol) added dropwise, followed by the careful addition of 2 mL of concentrated H_2_SO_4_ along the walls of the test tube. The formation of a purple ring at the interface indicated a positive result [17].

#### 2.5.3. Coumarins

For coumarin detection, 2 mg of each extract was dissolved in 2 mL of methanol, and a 10% NaOH solution was added dropwise. A positive reaction was indicated by the appearance of a yellow coloration, which disappeared upon subsequent acidification of the solution [17].

#### 2.5.4. Unsaturations

Unsaturated compounds were detected by dissolving 1 mg of each extract in 2 mL of methanol and adding four drops of a 2% aqueous KMnO_4_ solution. A positive result was indicated by discoloration of the solution or the formation of a brown precipitate, corresponding to MnO_2_ formation [17].

#### 2.5.5. Flavonoids

To evaluate the presence of flavonoids, 1 mg of each extract was dissolved in 2 mL of concentrated H_2_SO_4_. Color development was interpreted as follows: yellow coloration indicated flavonoids, orange–cherry coloration suggested flavones, red–bluish coloration corresponded to chalcones, and red–purple coloration was indicative of quinones [17].

#### 2.5.6. Quinones

Between 5 and 10 mg of each sample was mixed with 0.2 mL of ethanol and 0.4 mL of a 5% aqueous NaOH solution in a test tube. Color development was visually assessed, and the ultraviolet absorption spectrum of the resulting solution was recorded to confirm the presence of quinones [17].

#### 2.5.7. Saponins

An aqueous 10% NaHCO_3_ solution was prepared prior to analysis. Then, 2 mg of each extract was dissolved in 2 mL of methanol, followed by the addition of four drops of concentrated H_2_SO_4_. After gentle stirring, four drops of the NaHCO_3_ solution were added. The formation of persistent bubbles lasting longer than 1 min was considered indicative of saponins [17].

#### 2.5.8. Sesquiterpene Lactones

For sesquiterpene lactone detection, 2 mg of each extract was dissolved in 2 mL of ethanol, and three drops of a freshly prepared mixed reagent were added. A positive reaction was indicated by a color change from orange to dark red. The mixed reagent (1:1, *v*/*v*) consisted of Solution A (1% C_6_H_3_N_3_O_7_ in ethanol) and Solution B (10% NaOH) [17].

#### 2.5.9. Sterols and Terpenes

Each extract (1 mg) was dissolved in 2 mL of chloroform, followed by the careful addition of 2 mL of concentrated H_2_SO_4_. The formation of a red–brown ring at the interface was considered a positive reaction for sterols and methyl sterols [17].

#### 2.5.10. Phenolic Compounds (Tannins)

Phenolic compounds were evaluated by dissolving 1 mg of each extract in 2 mL of methanol and adding four drops of a 2.5% aqueous FeCl_3_ solution. The formation of a red, blue–violet, or green precipitate indicated a positive result for tannins [17].

### 2.6. Characterization of the Metabolic Profile of the Methanol Extract

The metabolomic profiling of the methanolic extract of *Scenedesmus* sp. was performed by the Centre for Metabolomics Innovation (Edmonton, Alberta, Canada). A total of 100 μg of the extract was analyzed using liquid chromatography coupled to high-resolution mass spectrometry (LC-HRMS). Chromatographic separation was carried out on a reverse-phase C18 column (2.1 × 100 mm, 1.7 μm; Waters Acquity UPLC BEH) using a linear water–acetonitrile gradient, both solvents containing 0.1% formic acid, which enabled the sequential elution of metabolites according to their polarity, from more polar to more hydrophobic compounds, following TMIC standardized protocols. Mass spectra were acquired in both positive and negative ionization modes using a Q-Exactive Orbitrap mass spectrometer (Thermo Fisher Scientific, Waltham, Massachusetts, EE. UU.) equipped with a heated electrospray ionization (HESI) source, operating at a resolution of 70,000 FWHM. Data processing, including peak detection, deconvolution, and retention time alignment, was performed using Compound Discoverer v3.3 (Thermo Fisher Scientific) with a mass tolerance of 5 ppm. Putative metabolite identification was achieved by matching accurate mass, isotopic patterns, retention times, and MS/MS fragmentation spectra against the HMDB, METLIN, and MassBank databases. Relative abundances (%) were calculated from normalized peak areas, and only metabolites with a relative abundance ≥ 1% were included in the main table, whereas those below this threshold are reported in Table S1 [18].

### 2.7. Cell Line

Murine L5178Y-R lymphoma cells (ATCC CRL-1722) were employed as the experimental tumor model, while human peripheral blood mononuclear cells were used as non-malignant controls. PBMCs were isolated from 20 mL of venous blood collected from a healthy volunteer, diluted tenfold in phosphate-buffered saline (PBS), and carefully layered onto 15 mL of Ficoll–Paque PLUS (GE Healthcare Life Sciences, Pittsburgh, PA, USA) to preserve phase separation. Density-gradient centrifugation was performed at 400 rpm for 30 min at 20 °C, after which the supernatant was discarded.

Both cell populations were maintained in RPMI-1640 medium (Life Technologies, Inc., Grand Island, NY, USA) supplemented with 10% heat-inactivated fetal bovine serum and 1% antibiotic–antimycotic solution. Cultures were incubated under controlled conditions at 37 °C in a humidified atmosphere containing 5% CO_2_.

### 2.8. In Vitro Antitumor Activity

L5178Y-R lymphoma cells were seeded at a density of 1 × 10^4^ cells per well, while PBMCs were plated at 1 × 10^5^ cells per well, using 100 µL of complete RPMI-1640 medium in flat-bottom 96-well plates (Corning Incorporated, Corning, NY, USA). After an initial 24 h stabilization period, cells were exposed in triplicate to graded concentrations of the extracts prepared by twofold serial dilution from a 1 mg/mL stock solution, generating final concentrations between 15.625 and 250 µg/mL in a total assay volume of 200 µL. Treatments were maintained for 48 h at 37 °C under a humidified atmosphere containing 5% CO_2_. Cellular metabolic activity was quantified through the MTT reduction assay (3-[4,5-dimethylthiazol-2-yl]-2,5-diphenyltetrazolium bromide; Affymetrix, Cleveland, OH, USA). Briefly, 15 µL of MTT solution was added to each well to reach a final concentration of 0.5 mg/mL, followed by incubation for 3 h at 37 °C. The resulting formazan precipitates were solubilized with dimethyl sulfoxide (DMSO; Sigma-Aldrich, Burlington, Massachusetts, EE. UU.), an using a MULTISKAN GO microplate reader (Thermo Fisher Scientific, Waltham, MA, USA). The development of a purple color was indicative of metabolically active cells [19].

Growth inhibition was calculated according to the formula % growth inhibition = 100 − ((OD_570_ in extract-treated cells/OD_570_ in untreated cells) × 100), with vincristine sulfate (0.05 µg/mL; Hospira, Warwickshire, UK) employed as the reference cytotoxic agent. Dose–response curves were generated by plotting log-transformed extract concentrations against inhibition percentages to estimate IC_50_ values. These values were subsequently used to determine the selectivity index (SI) by dividing the IC_50_ obtained for non-tumor cells by that of the lymphoma cells [20].

### 2.9. Antioxidant Activity Assay

#### 2.9.1. Extract

Free radical scavenging capacity of *Scenedesmus* sp. extracts was assessed using the 2,2-diphenyl-1-picrylhydrazyl (DPPH; Sigma-Aldrich) assay, following the general approach described by Rumpf et al. (2023) [21]. Dimethyl sulfoxide (DMSO) served as the solvent control, while ascorbic acid was included as the reference antioxidant within a concentration range of 10–100 µg/mL. Assays were conducted in 96-well microplates by combining equal volumes (100 µL) of DPPH solution and extract dilutions. Reaction mixtures were maintained at ambient temperature for 30 min under light-protected conditions. Absorbance was subsequently measured at 517 nm using a MULTISKAN GO microplate reader (Thermo Fisher Scientific). The percentage of DPPH radical neutralization was determined using Equation (1) [22].

(1) % DPPH= [(OD Neg control − OD Treatment)/(OD Neg control)] × 100

#### 2.9.2. Serum

We performed the DPPH assay to evaluate the antioxidant activity of the extract in serum. For this, we incubated 100 µL of the extract at different concentrations, 20 μL of serum, 10 mM sodium phosphate buffer with a pH of 7.4 (400 μL total volume), and 400 μL of a methanol solution of 0.1 mM DPPH at 21 °C for 30 min, and ODs were then measured at 520 nm, using Formula (1) to calculate the antioxidant activity [23].

#### 2.9.3. Hemolytic and Anti-Hemolytic Activity

Erythrocyte membrane integrity and resistance to oxidative damage were examined using standardized assays [24], with procedural modifications. Peripheral blood (20 mL) from a healthy donor was collected into EDTA-treated tubes. Red blood cells were isolated by repeated washing (three cycles) with phosphate-buffered saline (PBS, pH 7.2) and adjusted to a final concentration of 5% (*v*/*v*) in sterile PBS.

To assess hemolytic effects, erythrocyte suspensions were combined with increasing concentrations of the extracts (15.625–250 µg/mL) and incubated at 37 °C for 30 min in triplicate. Samples were subsequently centrifuged at 13,000 rpm for 5 min at 4 °C. Ultrapure water was employed to induce complete hemolysis, whereas PBS served as the non-hemolytic reference. The capacity of the extracts to prevent oxidative erythrocyte damage was analyzed by exposing the cell suspension to 150 mM 2,2′-azobis(2-amidinopropane) dihydrochloride (AAPH; Sigma-Aldrich) in the presence of the extracts. Incubations were conducted at 37 °C for 5 h under continuous shaking (200 rpm). Control groups included erythrocytes in PBS (negative control) and erythrocytes challenged with AAPH alone (oxidative control). After incubation, samples were centrifuged using the same parameters described above. In both experimental setups, aliquots (200 µL) of the supernatants were transferred to 96-well plates, and absorbance values were measured at 540 nm with a microplate spectrophotometer. Hemolysis and hemolysis inhibition percentages were calculated according to Equations (2) and (3):

(2) % Hemolysis = [(OD treatment)/(OD Positive control)] × 100

(3) % Anti-hemolytic Activity = 1 − [(OD treatment)/(OD Positive control)] × 100

### 2.10. Animals

Male BALB/c mice (6–7 weeks old) were obtained from the animal facility of the Immunology and Virology Laboratory, School of Biological Sciences, Autonomous University of Nuevo León (UANL), Mexico. Animals were maintained in individually ventilated cages equipped with environmental enrichment (cardboard tubes), with unrestricted access to standard chow and water. Housing conditions were carefully controlled to ensure a pathogen-free, low-stress environment, with temperature maintained at 22 °C, relative humidity at 45%, and a 12 h light/12 h dark photoperiod. All experimental procedures complied with internationally accepted standards for animal welfare in oncology research. Humane endpoints were established using a clinical monitoring system based on changes in body mass, fur appearance, posture, and spontaneous activity.

### 2.11. In Vivo Toxicity Assay and Hepatotoxicity of Extracts

An in vivo toxicity assay was performed following previously described methodologies [25]. For formulation, the extract was incorporated into microspheres by blending with 5 mg of food-grade calcium alginate (Molecular Cuisine Supplies, Mexico City, Mexico), which served as the delivery vehicle. Animals were assigned to treatment groups receiving oral doses of 250, 500, or 1000 mg/kg, administered over seven days, with 24 h intervals between doses. Animals were monitored for a total of 10 days, during which body weight and clinical condition were systematically recorded. Humane endpoints were predefined, and animals exhibiting a body weight loss of 20% or more, or reaching a clinical score of 3 or higher, were euthanized. For terminal procedures, anesthesia was induced by intraperitoneal injection of sodium pentobarbital at doses ranging from 25 to 40 mg/kg (Aranda Salud Animal, Querétaro, Mexico). Blood was collected via terminal cardiac puncture, after which euthanasia was completed. Samples were placed in anticoagulant-free tubes and centrifuged at 3000 rpm for 5 min to obtain serum, which was then used for biochemical analysis of hepatic function [26].

### 2.12. Glucose Tolerance Curve Test

To evaluate glucose tolerance, mice were fasted for 6 h. Basal blood glucose was measured from blood drawn from the mice’s tails, followed by oral administration of the methanol extract of *Scenedesmus* sp. at doses of 1000, 500, and 250 mg/kg, as well as calcium alginate at 5 mg/kg as the control. Subsequently, a 20% glucose solution adjusted to their weight was administered intraperitoneally. The initial glucose solution was prepared at a concentration of 200 mg/mL to deliver 0.1 mL per 10 g of mouse weight. After glucose administration, blood glucose levels were measured at 15, 30, 60, and 120 min to determine the area under the glucose measurement curve [27]. Blood glucose was measured using a True Metrix glucometer (Trividia Health, Fort Lauderdale, FL, USA).

### 2.13. Statistical Analysis

Data are presented as mean values ± standard deviation, derived from three technical replicates per treatment in in vitro assays and from groups of five animals per condition in in vivo experiments, each repeated independently three times. Statistical analysis was performed using GraphPad Prism version 9 (GraphPad Software Inc., San Diego, CA, USA). Survival outcomes were evaluated using Kaplan–Meier curves with comparisons made via the log-rank test. Changes in body weight were analyzed by two-way analysis of variance (ANOVA) for normally distributed data, followed by Tukey’s post hoc test to identify differences between groups. Tumor volume measurements were assessed using one-way ANOVA under the assumption of normality, with Dunnett’s multiple comparison test applied to compare treated groups to the control group. Statistical significance was set at *p* < 0.05.

## 3. Results

### 3.1. Antitumor and Biological Activity of Scenedesmus sp.

A yield of 6.2% was obtained from the methanolic extract of *Scenedesmus* sp. The extract exhibited an IC_50_ value of 168 ± 0.332 µg/mL against L5178Y-R murine lymphoma cells. In contrast, growth inhibition in normal human PBMCs was observed only at concentrations above 1500 µg/mL, resulting in a selectivity index (SI) of 8.928, which indicates high antitumor potency with low cytotoxicity toward normal cells. Regarding antioxidant activity, the extract showed an IC_50_ value of 237 µg/mL in the DPPH assay. No hemolytic effect was detected at the tested concentrations, with a calculated IC_50_ of 3019 µg/mL. In contrast, antihemolytic activity was observed with an IC_50_ of 199.3 µg/mL (Table 1).

### 3.2. In Vivo Toxicity Assay

**Figure 1:**
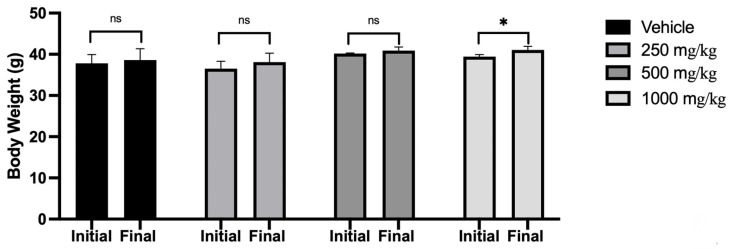
Mice body weight before and after the oral administration of *Scenedesmus* sp. methanol extract (ns, no significant difference; *, *p* < 0.05). Data represent the mean ± SD of five mice per experimental group from three independent experiments.

From the in vitro results, we selected the administration of 250 mg/kg, 500 mg/kg, and 1000 mg/kg oral doses of *Scenedesmus* sp. methanol extract for the in vivo study. Figure 1 shows an average increase of 2% in body weight in the vehicle group, 4% for the 250 mg/kg group, 2% in the 500 mg/kg group, and 3.9% in the 1000 mg/kg group at day 7 of the protocol.

We did not find significant alterations of serum albumin, total protein, globulin, total bilirubin, aspartate aminotransferase, alanine aminotransferase, and alkaline phosphatase by *Scenedesmus* sp. methanol extract, as compared with plasma control values from extract-untreated animals (negative control; Table 2).

### 3.3. Antioxidant and Hypoglycemic Effects of Scenedesmus sp.

**Figure 2:**
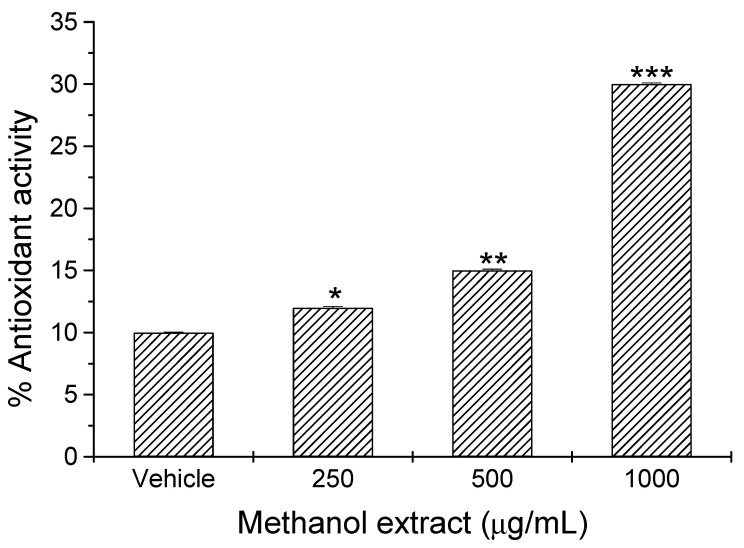
Antioxidant activity of *Scenedesmus* sp. methanol extract. Data represent the mean ± SD of five mice per experimental group from three independent experiments (*, *p* < 0.05; **, *p* < 0.02; ***, *p* < 0.001).

The evaluation of antioxidant potential was performed by measuring the elimination of free radicals using the 2,2-diphenyl-1-picrylhydrazyl (DPPH) reduction assay. We observed a significant (*p* < 0.05) concentration-dependent antioxidant activity of orally administered *Scenedesmus* sp. methanol extract (Figure 2). We found significant increases of 20% (*p* < 0.05), 50% (*p* < 0.01), and 200% (*p* < 0.001) in antioxidant activity of *Scenedesmus* sp. methanol extracts at respective concentrations of 250 mg/kg, 500 mg/kg, and 1000 mg/kg, as compared with that of the vehicle (Figure 2).

**Figure 3:**
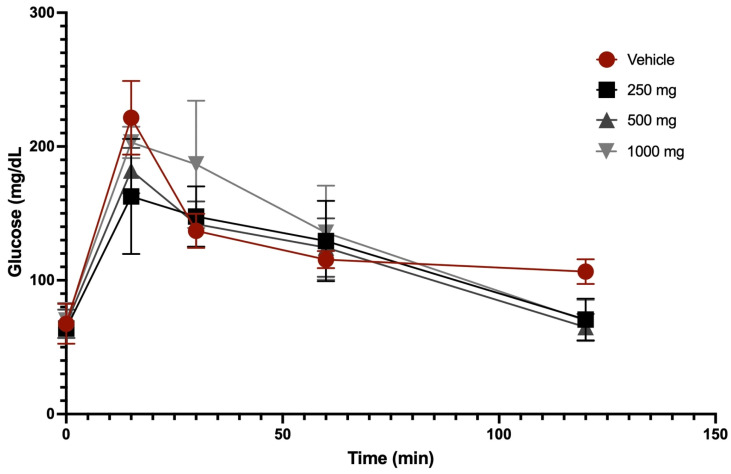
Glucose tolerance curves after oral administration of vehicle and *Scenedesmus* sp. methanol extract.

A glucose tolerance curve was obtained after the oral administration of 250 mg/kg, 500 mg/kg, and 1000 mg/kg of extract and vehicle in fasting animals. Figure 3 shows the concentration-dependent glucose tolerance curve after oral administration of *Scenedesmus* sp. methanol extract. The highest glucose values for the vehicle and extract treatments were observed at 15 min, where respective average values for vehicle, 250 mg/kg, 500 mg/kg, and 1000 mg/kg were 221.5 mg/dL, 159 mg/dL, 182 mg/dL, and 203 mg/dL. After 120 min, all groups returned to baseline glucose levels, except for the vehicle group, whose final glucose level of 106.5 mg/dL was higher than the initial value of 67.5 mg/dL (Figure 3).

### 3.4. Phytochemical Profile

The phytochemical characterization of *Scenedesmus* sp. methanol extract allowed for the identification of diverse compounds. Results of the analysis showed the presence of alkaloids, tannins, triterpenes, protein-like molecules, and the absence of chemical groups such as flavonoids, saponins, steroids, and reducing sugars (Table 3).

### 3.5. Metabolomic Profile

The metabolomic analysis of the methanolic extract of *Scenedesmus* sp. identified a total of 183 primary metabolites out of 630 features available in the database, of which nineteen exhibited a relative abundance ≥ 1% (Table 4). Amino acids constituted the dominant fraction of the chemical profile, representing the largest proportion of the identified compounds. Valine was the most abundant metabolite (12.0%), followed by lactic acid (11.71%), choline (11.33%), and alanine (10.68%), all exceeding 10% relative abundance. Together, these four metabolites accounted for more than 45% of the total metabolite content, highlighting their importance in the biochemical composition of the extract.

Hexose-type carbohydrates represented another major group, contributing 7.68% of the total abundance. Metabolites of intermediate abundance included threonine (5.37%) and glycine (5.33%), further reinforcing the predominance of amino acids in the extract. Other compounds present at moderate levels were betaine (3.70%), glutamic acid (3.41%), β-hydroxybutyric acid (3.09%), glyceric acid (2.88%), tyrosine (2.70%), succinic acid (2.41%), and proline (2.05%). In addition, several organic acids and amino acid derivatives—such as aspartic acid (1.62%), asparagine (1.53%), serine (1.43%), malic acid (1.42%), and phenylalanine (1.09%)—were detected slightly above the established 1% threshold.

Overall, the metabolite composition reflects high activity of central carbon metabolism, amino acid biosynthesis, and osmoprotective pathways, which are characteristic features of microalgal systems. Metabolites detected at relative abundances < 1% were excluded from the main table to facilitate interpretation; however, overall, they represented 8.58% of the metabolite pool and are fully documented in Table S1.

## 4. Discussion

A nutraceutical is defined as a dietary supplement—typically delivered in a non-food matrix such as capsules, tablets, or powders—containing a concentrated bioactive natural compound originally present in food sources and capable of providing benefits in the prevention or treatment of disease [29]. Microalgae are widely consumed as nutritional supplements due to the health-promoting properties of their bioactive metabolites, as is the case with *Scenedesmus* sp. The metabolomic profiling of the methanolic extract revealed a composition rich in amino acids, organic acids, osmolytes, and carbohydrates, indicating high nutraceutical and bioactive potential. The accumulation of amino acids, sugars, and organic acids (such as succinic, malic, and lactic acids) suggests that the extract contains intermediates of central metabolism, which may support its use as a nutraceutical supplement by contributing metabolic precursors, antioxidants, osmotic regulators, and energy-related molecules.

The antioxidant effect can be largely attributed to the presence of amino acids, organic acids, phenolic compounds, and carotenoids in the *Scenedesmus* extract, all of which are well known for their capacity to neutralize reactive oxygen species (ROS), maintain cellular redox homeostasis, and serve as biosynthetic precursors and nutritional support [11]. This combination of metabolites makes the extract of *Scenedesmus* sp. a promising candidate both as a nutraceutical supplement (providing amino acids, osmolytes, and metabolic intermediates) and as a source of compounds with antitumor potential through mechanisms involving oxidative stress modulation, epigenetic regulation, apoptosis induction, and metabolic reprogramming.

Redox modulation has been documented in previous studies, where dietary supplementation with *Scenedesmus* sp. in male C57BL/6 mice significantly altered redox homeostasis in a dose-dependent manner. Notably, a 5% inclusion of microalgae in the diet was positively correlated with increased levels of reduced glutathione (GSH), indicating activation of endogenous antioxidant defenses [30]. Consistent with these findings, our study demonstrated an increase in serum antioxidant potential in mice treated with the extract at different doses, showing free radical inhibition of 31.3% at 1000 mg/kg, 15.2% at 500 mg/kg, and 12.1% at 250 mg/kg. These observations support the hypothesis that the bioactive compounds present in *Scenedesmus* sp. effectively modulate systemic redox status, possibly through the induction of antioxidant enzymes or increased synthesis of metabolites such as GSH [30].

The metabolomic composition also supports the hypothesis that the antitumor effect observed for the extract may originate from a synergistic interaction among several metabolites: osmolytes and methyl donors (betaine 3.70%, choline 11.33%), which modulate methylation, cellular protection, and oxidative stress; and epigenetically active metabolites such as β-hydroxybutyrate (β-HB), capable of inhibiting histone deacetylases (HDACs), altering histone acetylation, activating tumor-suppressor pathways, and promoting apoptosis or senescence. Betaine—an established methyl donor and osmoprotectant—has been shown in multiple studies to reduce oxidative stress, modulate inflammation and apoptosis, and enhance autophagy and hepatoprotection under metabolic stress. Studies in DU-145 prostate cancer cells have demonstrated dose-dependent cytotoxicity mediated by increased oxidative stress, inflammation, caspase activation, and apoptosis induction [31,32]. This dual behavior (cytoprotection in healthy cells and cytotoxicity in cancer cells) may partially explain the antitumor effect shown by our methanolic extract: an adequate concentration of betaine, in combination with other metabolites, may create a cellular environment that favors apoptosis in tumor cells while supporting normal cell metabolism [33].

A key metabolite of interest is β-hydroxybutyrate (β-HB), which was detected in the extract. Beyond serving as an energy substrate during fasting or ketosis, β-HB functions as a signaling molecule that regulates oxidative stress, inflammation, cellular protection, and epigenetic processes [34]. In particular, the inhibition of HDACs mediated by β-HB has been documented, promoting beneficial epigenetic remodeling, such as increased histone acetylation; activation of tumor-suppressor genes; and modulation of oxidative stress, inflammation, and cellular metabolism [34,35]. In liver carcinogenesis models, short-chain fatty acids, structurally like butyrate—and by analogy β-HB—have been associated with decreased HDAC activity, induction of apoptosis, telomerase suppression, oncogene inhibition, and reduction in preneoplastic lesions [36,37,38]. These findings reinforce the hypothesis that β-HB present in the *Scenedesmus* extract may contribute, at least in part, to the antitumor activity observed in vitro. Other studies using *Scenedesmus* sp. have reported growth inhibition of the L5178Y-R lymphoma cell line, with an IC_50_ of 362.9 ± 13.5 µg/mL, similar to our findings, where we observed an IC_50_ of 168 ± 0.332 µg/mL—approximately 50% lower, indicating higher potency of our extract [13]. These differences may stem from the fact that microalgal metabolism is highly responsive to external factors such as temperature, pH, aeration, and nutrient composition. In another study by Marrez et al. (2019) using *Scenedesmus obliquus*, significant antitumor activity was also observed against HCT116 and HepG2 cell lines, with IC_50_ values of 25.6 and 42.7 µg/mL, respectively, proving the effectiveness of *Scenedesmus* strains beyond lymphoma models [39]. As previously noted, these cytotoxic effects are often associated with their high content of fatty acids, lipids (49.7%), and phenolic compounds, which have been shown to exert antiproliferative activity through apoptosis induction [40].

A major limitation when evaluating bioactive compounds from natural sources is the limited evidence regarding their safety, particularly concerning potential hepatotoxicity. Microalgae used in dietary supplements have shown a favorable biosafety profile in different experimental models. Da Silva et al. (2020) demonstrated that supplementation with lyophilized microalgae at doses of 11.6 and 23.2 g/100 g of feed did not alter liver function, as evidenced by unchanged serum AST and ALT levels [14]. Consistently, our study found no significant differences in hepatic function markers between treated groups and controls, suggesting that the evaluated doses of *Scenedesmus* sp. extract do not induce acute liver toxicity in this experimental model. These results reinforce the safety of microalgae as a source of functional compounds for nutraceutical applications. Regarding glucose regulation, Da Silva et al. (2020) also reported that higher microalgae concentrations in the diet resulted in a 42% reduction in serum glucose (128.7 mg/dL vs. 223.3 mg/dL in controls) [14]. A similar trend was observed in our study: although baseline glucose levels were comparable among groups (control: 67.5 mg/dL; 250 mg/kg: 64.0 mg/dL; 500 mg/kg: 67.5 mg/dL; 1000 mg/kg: 70 mg/dL), the area under the glucose curve over 120 min was markedly lower in extract-treated groups, with levels returning close to baseline, except in the control group (final glucose: 106.5 mg/dL). These findings are consistent with the hypoglycemic effects reported for *Scenedesmus* and suggest that its metabolites may exert glycemic control through antioxidant, anti-inflammatory, or metabolic modulation mechanisms [14]. This effect on glycemic homeostasis may result from the synergistic action of carotenoids, pigments, chlorophyll, phenolics, polysaccharides, dietary fiber, and unsaturated fatty acids—bioactive compounds previously associated with beneficial metabolic outcomes [41]. These metabolites may influence insulin sensitivity, intestinal glucose absorption, and oxidative stress related to metabolic dysfunction.

## 5. Conclusions

The present study demonstrates that the methanolic extract of *Scenedesmus* sp. exhibits a complex and diverse chemical profile, which likely underlies its antioxidant, antitumoral, and glucose metabolism-modulating activities observed in vitro and in vivo. The integration of metabolomic, biological, and functional data supports the potential of *Scenedesmus* sp. as a promising source of bioactive compounds with nutraceutical and pharmacological relevance. These findings position this microalga as a valuable candidate for further development; however, additional studies are warranted to elucidate the molecular mechanisms involved, define the contribution of key metabolites, and validate its therapeutic efficacy and safety in advanced preclinical and clinical models.

## Figures and Tables

**Table 1:** Yield, antitumor, and biological activities of *Scenedesmus* sp. BS1135 methanol extract.

Extract	Yield (%)	L5178Y-R IC_50_ ^1^	PBMC IC_50_ ^1^	Selectivity Index	Antioxidant Activity ^1^	Hemolysis ^1^	Anti-Hemolysis ^1^
Methanol	6.2	168 ± 0.332	>1500	8.928	237.3 ± 0.332	3029 ± 0.536	199.3 ± 0.341

^1^ IC_50_ in µg/mL. Data represent the mean ± SD of three replicate determinations per treatment per experimental group from three independent experiments. Vincristine was used as a positive control, causing 80% lymphoma cell growth inhibition.

**Table 2:** Biochemical values and liver function test.

Parameter	250 mg/kg Treatment	500 mg/kg Treatment	1000 mg/kg Treatment	Vehicle	Negative Control	Reference Values	References
Albumin	3.9 ± 0.20	2.63 ± 0.49	2.87 ± 0.31	3.0 ± 0.07	2.74 ± 0.21	2.0–4.6 g/dL	[28]
Total protein	3.4 ± 0.21	4.60 ± 0.70	4.73 ± 0.15	4.2 ± 0.14	4.14 ± 0.19	4.3–6.4 g/dL	[28]
Total bilirubin	0.11 ± 0.01	0.12 ± 0.01	0.15 ± 0.05	0.1 ± 0.01	0.09 ± 0.08	0.3–0.8 mg/dL	[28]
Aspartate aminotransferase	158.8 ± 119.9	118.0 ± 29.0	140.07 ± 45.4	111.4 ± 0.92	173 ± 32.75	69–191 U/L	[28]
Alanine aminotransferase	72.0 ± 10.9	41.0 ± 12.1	49.20 ± 13.5	43.5 ± 1.9	40.54 ± 6.85	26–120 U/L	[28]
Alkaline phosphatase	70.0 ± 8.1	42.0 ± 32.0	68.23 ± 36.1	66.5 ± 2.1	36.8 ± 14.67	44–118 U/L	[28]

Data represent the mean ± SD of five mice per experimental group from three independent experiments.

**Table 3:** Phytochemical analysis of *Scenedesmus* sp. methanol extract.

Class	Methanol Extract
Alkaloids	+ ^1^
Flavonoids	−
Tannins	+
Triterpenes	+
Proteins	+
Saponins	−
Steroids	−
Reducing sugars	−

^1^ +, presence; −, absence.

**Table 4:** Major metabolites identified in the methanolic extract of *Scenedesmus* sp. by untargeted metabolomic analysis.

Metabolite	Molecular Formula	Chemical Class	µM	Relative Abundance (%)
Valine	C_5_H_11_NO_2_	Essential amino acid, aliphatic	6260.0000	12.000
Lactic acid	C_3_H_7_NO_2_	Nonessential amino acid	6110.0000	11.712
Choline	C_4_H_9_NO_3_	Essential amino acid, polar	5910.0000	11.329
Alanine	C_2_H_5_NO_2_	Nonessential amino acid	5570.0000	10.677
Hexose	C_5_H_9_NO_4_	Acidic amino acid	4005.6862	7.678
Threonine	C_9_H_11_NO_3_	Aromatic amino acid	2800.0000	5.367
Glycine	C_5_H_9_NO_2_	Cyclic amino acid	2780.0000	5.329
Betaine	C_4_H_7_NO_4_	Acidic amino acid	1930.0000	3.700
Glutamic acid	C_4_H_8_N_2_O_3_	Polar amino acid	1780.0000	3.412
beta-Hydroxybutyric acid	C_3_H_7_NO_3_	Polar amino acid	1610.0000	3.086
Glyceric acid	C_9_H_11_NO_2_	Essential aromatic amino acid	1500.0000	2.875
Tyrosine	C_3_H_6_O_3_	Organic acid	1410.0000	2.703
Succinic acid	C_4_H_8_O_3_	Ketone body	1260.0000	2.415
Proline	C_3_H_6_O_4_	Organic acid	1070.0000	2.051
Aspartic acid	C_4_H_6_O_4_	Dicarboxylic acid	846.0000	1.622
Asparagine	C_4_H_6_O_5_	Dicarboxylic acid	800.0000	1.533
Serine	C_6_H_12_O_6_	Carbohydrate	745.0000	1.428
Malic acid	C_5_H_14_NO	Amino alcohol	738.0000	1.415
Phenylalanine	C_5_H_11_NO_2_	Osmolite	566.0000	1.085
Other	N/A	N/A	4478.051	8.584

Only metabolites with a relative abundance ≥ 1% are shown. Compounds detected at <1% were omitted from this table for clarity but are fully listed in Table S1. N/A: not applicable.

## Data Availability

The original contributions presented in this study are included in the article. Further inquiries can be directed to the corresponding authors.

## Supplementary Materials

The following supporting information can be downloaded at: https://www.mdpi.com/article/10.3390/foods15020186/s1, Table S1. Metabolites identified in the methanolic extract of *Scenedesmus* sp. by metabolomic analysis in abundance less than 1%.
