# Supplementary material for: Nutraceutical and Antitumoral Potential of Scenedesmus sp. in In Vitro and In Vivo Models
Source: Foods. 2026 Jan 6;15(2):186. doi: 10.3390/foods15020186 (PMC12840518; doi:10.3390/foods15020186)
Supplement: Supplementary file 1 [file foods-15-00186-s001.zip › foods-4045404-supplementary.pdf]

Table S1. Metabolites identified in the methanolic extract of Scenedesmus sp. by metabolomic analysis in abundance less than 1%.

Metabolite	μM	Relative Abundance (%)	Metabolite	μM	Relative Abundance (%)
Lysine	382.0000	0.732	PC ae C34:0	4.6505	0.009
Benzoic acid	371.0000	0.711	Methylmalonic acid	4.5700	0.009
Methionine-sulfoxide	353.0000	0.677	PC aa C38:0	3.7491	0.007
Isoleucine	323.0000	0.619	PC ae C36:4	3.4883	0.007
Oxalic acid	280.0000	0.537	PC ae C34:3	3.0963	0.006
Tryptophan	242.0000	0.464	PC ae C40:1	2.8533	0.005
Glutaric acid	167.0000	0.320	Diacetylspermine	2.8500	0.005
para-Hydroxyphenylacetic acid	158.0000	0.303	PC ae C38:0	2.5528	0.005
Arginine	140.0000	0.268	PC ae C38:5	2.4272	0.005
PC aa C36:2	139.7769	0.268	PC ae C38:6	2.1195	0.004
Leucine	127.0000	0.243	PC ae C36:5	1.9860	0.004
Tyramine	126.0000	0.242	LysoPC a C17:0	1.9450	0.004
Proline-Betaine	111.0000	0.213	PC aa C38:4	1.8492	0.004
LysoPC a C18:2	102.9436	0.197	LysoPC a C14:0	1.7394	0.003
Ornithine	102.0000	0.196	Methionine	1.6700	0.003
Putrescine	91.4000	0.175	PC aa C38:5	1.5129	0.003
PC aa C34:2	74.1852	0.142	PC ae C40:2	1.4549	0.003
Histidine	71.8000	0.138	LysoPC a C28:0	1.2467	0.002
Citrulline	69.2000	0.133	PC ae C32:2	1.1695	0.002
trans-Hydroxyproline	65.0000	0.125	PC ae C32:1	1.1522	0.002
LysoPC a C18:1	62.0293	0.119	PC aa C38:6	1.1421	0.002
PC aa C34:3	52.8291	0.101	LysoPC a C28:1	1.1319	0.002
Propionic acid	50.3000	0.096	PC aa C30:0	1.1025	0.002
PC aa C36:3	44.8562	0.086	PC ae C40:6	1.0740	0.002
PC aa C36:4	40.4017	0.077	PC ae C30:0	0.8779	0.002
PC aa C34:1	37.7656	0.072	C12:1	0.8276	0.002
PC aa C36:5	35.7662	0.069	C2	0.7703	0.001
PC aa C36:1	33.1391	0.064	PC aa C40:1	0.7512	0.001
PC ae C38:2	33.0576	0.063	PC ae C40:5	0.7043	0.001
C0	31.2071	0.060	PC ae C30:1	0.7003	0.001
Total dimethylarginine	30.5000	0.058	PC aa C40:2	0.6948	0.001
Asymmetric dimethylarginine	28.0000	0.054	C3:1	0.6840	0.001
Citric acid	27.1000	0.052	PC aa C40:6	0.6523	0.001
LysoPC a C16:0	25.8227	0.049	LysoPC a C26:0	0.6505	0.001
Fumaric acid	23.1000	0.044	C10:1	0.6133	0.001
PC ae C36:1	21.8955	0.042	PC aa C28:1	0.6015	0.001
Pyruvic acid	17.9000	0.034	PC aa C30:2	0.5842	0.001
PC aa C32:2	17.4416	0.033	Serotonin	0.5760	0.001

alpha-Ketoglutaric acid	16.9000	0.032	LysoPC a C24:0	0.5357	0.001
PC aa C34:4	16.5726	0.032	C16:2	0.5299	0.001
PC aa C36:6	15.6589	0.030	PC ae C40:3	0.5227	0.001
cis-Hydroxyproline	14.4000	0.028	C12	0.5174	0.001
PC ae C36:2	14.3833	0.028	Jasmonic acid	0.5110	0.001
Trimethylamine N-oxide	14.3000	0.027	LysoPC a C26:1	0.4910	0.001
LysoPC a C16:1	14.2561	0.027	C16:2OH	0.4879	0.001
PC aa C36:0	12.6459	0.024	PC aa C42:2	0.4803	0.001
PC ae C38:1	11.5847	0.022	PC aa C40:5	0.4485	0.001
PC aa C32:1	11.0218	0.021	C16:1	0.4457	0.001
PC aa C32:0	10.9403	0.021	PC aa C42:1	0.4345	0.001
PC ae C38:3	10.7129	0.021	C16OH	0.4334	0.001
Phenylethylamine	10.6000	0.020	PC aa C40:3	0.4334	0.001
Indole acetic acid	10.0000	0.019	C10	0.3868	0.001
Taurine	9.7500	0.019	C18:2	0.3759	0.001
PC aa C38:3	8.9509	0.017	PC aa C42:6	0.3708	0.001
Glutamine	8.3200	0.016	PC ae C30:2	0.3664	0.001
p-Hydroxyhippuric acid	7.9600	0.015	PC ae C42:1	0.3641	0.001
PC aa C32:3	7.7284	0.015	C4:1	0.3608	0.001
PC aa C38:1	7.7250	0.015	PC ae C42:2	0.3530	0.001
LysoPC a C18:0	7.2062	0.014	C18:1	0.3462	0.001
Spermidine	7.0500	0.014	C16:1OH	0.3433	0.001
PC ae C36:0	6.3433	0.012	C3	0.3256	0.001
PC ae C36:3	5.1264	0.010	C16	0.3104	0.001
PC ae C34:1	4.9683	0.010	C3OH	0.2953	0.001
Aconitic acid	4.9200	0.009	PC ae C40:4	0.2904	0.001
Hippuric acid	4.8900	0.009	PC aa C40:4	0.2848	0.001
PC ae C34:2	4.8516	0.009	PC aa C42:5	0.2826	0.001
PC ae C38:4	4.8270	0.009	LysoPC a C20:4	0.2806	0.001
Salicylic acid	4.7200	0.009	PC aa C42:0	0.2698	0.001
